# Developing a clinical-radiomic prediction model for 3-year cancer-specific survival in lung cancer patients treated with stereotactic body radiation therapy

**DOI:** 10.1007/s00432-023-05536-x

**Published:** 2024-01-26

**Authors:** Bao-Tian Huang, Ying Wang, Pei-Xian Lin

**Affiliations:** 1 Department of Radiation Oncology, Cancer Hospital of Shantou University Medical College, Shantou, 515000 China; 2 Department of Nosocomial Infection Management, The Second Affiliated Hospital of Shantou University Medical College, Shantou, 515000 China

**Keywords:** Clinical-radiomic model, 3-year cancer-specific survival, Lung cancer, Stereotactic body radiation therapy

## Abstract

**Purpose:**

The study aims to develop and validate a combined model for predicting 3-year cancer-specific survival (CSS) in lung cancer patients treated with stereotactic body radiation therapy (SBRT) by integrating clinical and radiomic parameters.

**Methods:**

Clinical data and pre-treatment CT images were collected from 102 patients treated with lung SBRT. Multivariate logistic regression and the least absolute shrinkage and selection operator were used to determine the clinical and radiomic factors associated with 3-year CSS. Three prediction models were developed using clinical factors, radiomic factors, and a combination of both. The performance of the models was assessed using receiver operating characteristic curve and calibration curve. A nomogram was also created to visualize the 3-year CSS prediction.

**Results:**

With a 36-month follow-up, 40 patients (39.2%) died of lung cancer and 62 patients (60.8%) survived. Three clinical factors, including gender, clinical stage, and lymphocyte ratio, along with three radiomic features, were found to be independent factors correlated with 3-year CSS. The area under the curve values for the clinical, radiomic, and combined model were 0.839 (95% CI 0.735–0.914), 0.886 (95% CI 0.790–0.948), and 0.914 (95% CI 0.825–0.966) in the training cohort, and 0.757 (95% CI 0.580–0.887), 0.818 (95% CI 0.648–0.929), and 0.843 (95% CI 0.677–0.944) in the validation cohort, respectively. Additionally, the calibration curve demonstrated good calibration performance and the nomogram created from the combined model showed potential for clinical utility.

**Conclusion:**

A clinical-radiomic model was developed to predict the 3-year CSS for lung cancer patients treated with SBRT.

## Introduction

Lung cancer is the leading cause of cancer-related death worldwide (Leiter et al. 2023). For early-stage non-small-cell lung cancer (NSCLC) who are medically inoperable, stereotactic body radiation therapy (SBRT) is recommended as a standard treatment option (Ball et al. 2019; Safavi et al. 2021; Shintani et al. 2018; Vansteenkiste et al. 2014). The technique has been demonstrated to achieve effective local control (LC) rate by administering concentrated high dose to the tumor, while simultaneously minimizing the impact on nearby organs at risk (OARs) (Amini et al. 2014). Notably, multiple studies have reported comparable outcomes between SBRT and surgical interventions (Zhang et al. 2014; Zheng et al. 2014).

Although SBRT has achieved encouraging LC rates, the reported 3-year overall survival (OS) is only 54.2% (Baker et al. 2020). Predicting survival in this patient population has proven to be challenging (Klement et al. 2016; Young et al. 2017). Survival prediction at the individual patient level can assist radiation oncologists in treatment decision-making and provide an opportunity to add treatment for patients after SBRT (Baker et al. 2020; Jiao et al. 2021).

Recently, some predictors have been reported to be associated with OS after SBRT, such as cardiac dose (Tembhekar et al. 2017), mean lung dose (Dupic et al. 2020), biologically effective dose (BED) of the prescription and maximum dose (Stahl et al. 2016; Tateishi et al. 2021), pre-treatment FDG-PET standardized uptake values (Kocher et al. 2018), and pre-treatment hemoglobin level (Shaverdian et al. 2016). Radiomics, a non-invasive technology that converts medical images into a high-dimensional mineable feature space via high-throughput quantitative feature extraction (Bera et al. 2022; Gillies et al. 2016; Lambin et al. 2012, 2017; Reuze et al. 2018), has been introduced for the prediction of treatment responses, patient stratification, and prognosis for lung cancer patients in recent years (Chen et al. 2017, 2023; Constanzo et al. 2017; Coroller et al. 2015; Huang et al. 2016; Lee et al. 2017; Li et al. 2018; Mattonen et al. 2016). In particular, radiomic features extracted from CT images have shown promising performance in predicting OS in NSCLC patients treated with SBRT (Jiao et al. 2021; Li et al. 2018; Sawayanagi et al. 2022; Somasundaram et al. 2023; Starkov et al. 2019). However, most studies to date have employed OS as endpoint and there is a scarcity of studies that have integrated clinical and radiomic features to predict cancer-specific survival (CSS) in lung cancer patients undergoing SBRT.

Therefore, this study aims to develop and validate a combined model that integrates clinical and radiomic signatures for predicting 3-year CSS in lung cancer patients treated with SBRT.

## Methods

### Patient selection

This retrospective study followed the guidelines of the Declaration of Helsinki and was approved by the Ethics Committee of Cancer Hospital of Shantou University Medical College. Informed consent requirement was waived for the study. The study included a total of 102 patients diagnosed with primary lung cancer and treated with lung SBRT from January 2012 to March 2020. The inclusion criterion were as follows: (a) confirmed primary NSCLC treated with SBRT; (b) CT scan before the radiotherapy treatment; (c) availability of clinical characteristics and follow-up data. The exclusion criteria were: (a) secondary NSCLC; (b) incomplete radiotherapy treatment; (c) lack of clinical characteristics data; (d) follow-up less than 36 months.

### Radiotherapy treatment

All patients were immobilized in a custom-made mold with supine position. A Brilliance Big Bore CT scanner (Philips Brilliance CT Big Bore Oncology Configuration, Cleveland, OH, USA) was used with the patient’s arm elevated above the head. Tumor motion was accessed using four-dimensional computed tomography (4DCT) or three-dimensional computed tomography (3DCT). The gross tumor volume (GTV) was delineated within the lung window. For 4DCT images, ten phases of 4DCT datasets with respiratory motion information were integrated to form the internal target volume (ITV). For 3DCT images, some ITV were defined based on the GTV in peak-exhale and peak-inhale respiratory phases, while others were observed on the fluoroscopy to determine the tumor motion amplitude. The planning target volume (PTV) was generated by expanding the ITV with isotropic margins of 5 mm axially and 1 cm in the rostral-caudal direction. Fractional set-up error was corrected before each treatment using cone-beam CT equipped on the linear accelerator. The patients were randomly assigned to the training and validation groups in a 7:3 ratio. To compare the radiobiological effect of different fraction regimes, the BED was calculated using the formula derived from the linear quadratic (LQ) model, BED = *D* × (1 + *d*/*α*/*β*), where *D* is the nominal total dose, d is the fractional dose, and the *α*/*β* ratio is assigned to 10 Gy.

### Follow-up

Patient prognosis was evaluated through review of outpatient medical records, telephone consultations, and social security death indices. CT scans were performed every 3 months after the first year of treatment and 6 months thereafter. The primary endpoint of the study was the CSS outcome, which recorded the survival outcome from the day of the first treatment until the end of the 36-month follow-up caused by cancer-related death.

### Clinical parameter collection

We collected baseline clinical parameters for primary lung cancer before SBRT. This included gender, clinical stage, histology, GTV volume, PTV volume, maximum diameter for the tumor (MD), equivalent diameter for the tumor (ED), lymphocyte ratio (LCR), the maximum dose in PTV recorded as BED (BEDPTV_max_), fractional dose, and treatment duration.

### CT image acquisition and region of interest (ROI) segmentation

We acquired CT images of the patients using a Brilliance Big Bore CT scanner (voxel size: 1.0 × 1.0 × 3.0 mm, tube voltage: 120 kV, tube current: 350 mA, convolution kernel: Philips Healthcare’s B, reconstruction matrix: 512 × 512). The scan range was from the apex to the base of the lung. Subsequently, we transferred the CT images to Eclipse treatment planning system (Version 10.0, Varian Medical system, Inc., Palo Alto, CA) for tumor contouring, also known as ROI segmentation. This was performed by a radiologist with over 10 years of work experience.

### Radiomic feature extraction

We used an open-source Python package, PyRadiomics (version 3.6.0, https://github.com/Radiomics/pyradiomics), to automatically extract radiomics features from the ROIs. These features can be categorized into first order features, second-order features and higher-order features. First-order features are typically histogram-based and analyze the gray level signal intensity in a ROI, regardless of the spatial relationships between adjacent voxels. Examples of these features include uniformity, entropy, mean, median and kurtosis (Pyradiomics 2016). Second-order features, or “texture” features, examine the spatial relationship between gray-level signal intensities by constructing a gray-level dependence matrix, such as gray level co-occurrence matrix (GLCM), gray level dependence matrix (GLDM), gray level run length matrix (GLRLM), gray level size zone matrix (GLSZM) and neighborhood gray-tone difference matrix (NGTDM) (Pyradiomics 2016). To ensure the repeatability of our results, we performed resampling and *z* score normalization as preprocessing steps.

### Selection of radiomics features

To minimize differences between observers and enhance the robustness of features, two radiologists randomly selected 30 patients and delineated the tumor each. The intra-class correlation coefficient (ICC) was then calculated from the extracted features of these 30 patients to assess the intraobserver and interobserver reproducibility. Features with ICC > 0.75 were considered to have good agreement and were used for further analysis. The ICC analysis was conducted using the “irr” package in R studio (version 4.1.2, http://www.R-project.org; The R Foundation).

To avoid overfitting issues and reduce computation complexity, univariate analysis, least absolute shrinkage and selection operator (LASSO) with penalty parameter tuning conducted by tenfold cross-validation, and stepwise regression were employed to select significant radiomics features from the training group. The process of radiomic feature selection was performed in R studio. LASSO logistic regression was conducted using the “glmnet” package.

### Development of prediction models

To investigate the association of clinical and radiomic parameters with 3-year CSS for primary lung cancer after SBRT, three different prediction models were established, respectively. The clinical model was established using clinical variables, including dosimetric parameters. The radiomic model was developed using radiomic signatures. The combined model was constructed by combining clinical and radiomics variables.

### Evaluation of model performance

The performance of the prediction models was evaluated in terms of accuracy, sensitivity and specificity. The area under the curve (AUC) values on the receiver operating characteristic (ROC) curve was used to evaluate the discriminative ability of the models. ROC curves were drawn using MedCalc software (MedCalc, Version 20.015, MedCalc Software Ltd). Additionally, the Hosmer–Lemeshow test and calibration curve plotting the actual versus predicted probabilities were also employed to assess the calibration performance of the models.

### Construction of nomogram

A nomogram based on the combined model was created to visualize the 3-year CSS probability for lung cancer patients after SBRT. The nomogram was plotted using the “rms” package in R studio. The potential net benefit of the predictive models at different threshold probabilities was quantified, and the clinical usefulness was evaluated by the decision curve analysis (DCA) using the “dca.R” package in R studio.

### Statistical analysis

The optimal cut-off point for BEDPTV_max_ was determined by the Youden’s index on the ROC curve and then the continuous variable was translated into categorical variable. The Student *t* test or Mann–Whitney *U* test was used to compare continuous variables, while Chi-square test or Fisher’s exact test was applied for categorical variables between the training and validation group. A *p* value < 0.05 was considered statistically significant. Binary logistic regression was performed for both univariate and multivariate analyses to assess the relationship between risk factors and 3-year CSS. Variables with *p* value < 0.05 in the univariate analysis were entered into a stepwise multivariate logistic regression (method, forward: likelihood ratio) to estimate the *p* values and odd ratio (OR) values, with − 2 × Log-likelihood as the information criterion. To avoid the unstable and imprecise estimates of the coefficients, multicollinearity test was employed to exclude highly correlated variables before performing the multivariate logistic regression analysis. The tolerance and variance inflation factor (VIF) was used to evaluate multicollinearity among independent factors, with tolerance < 0.1 and a VIF > 10 between two factors indicative of multicollinearity (Marcoulides and Raykov 2019). To compare the performance of different models, the AUC values of the clinical model, radiomic model, and combined model were compared using the Delong test with *p* value < 0.05 indicative of statistically significant between two models. All the statistical analyses were performed in SPSS (version 25.0, IBM Corp., New York, NY; formerly SPSS Inc., Chicago, IL).

## Results

### Patients’ characteristics and survival outcomes

The study recruited a total of 102 patients (108 lesions) with primary lung cancer, with 74 lesions in the training group and 34 lesions in the validation group. Baseline characteristics of patients in the training and validation group were presented in Table 1. There were no significant different variables in baseline characteristics between the two groups, indicating a balance between the two sets of data. Within a 3-year follow-up, 40 patients (39.2%) died of lung cancer and 62 patient (60.8%) survived.

**Table 1:** Patients’ characteristics in the training and validation group

Characteristics	Overall (*n* = 108)	Training (*n* = 74)	Validation (*n* = 34)	*p* value
	Counts (%)/mean (range)	Counts (%)/mean (range)	Counts (%)/mean (range)	
Gender				1.00
Male	88 (81.5)	60 (81.1)	28 (82.3)	
Female	20 (18.5)	14 (18.9)	6 (17.7)	
Clinical stage				0.20
I	50 (46.3)	35 (47.3)	15 (44.2)	
II	12 (11.1)	5 (6.8)	7 (20.6)	
III	16 (14.8)	12 (16.2)	4 (11.8)	
IV	30 (27.8)	22 (29.7)	8 (23.4)	
Histology				0.42
Adenocarcinoma	51 (47.2)	35 (47.3)	16 (47.1)	
SCC	34 (31.5)	21 (28.4)	13 (38.2)	
Unknown	23 (21.3)	18 (24.3)	5 (14.7)	
GTV volume (cc)	46.6 (359.5)	45.6 (319.8)	48.7 (359.5)	0.83
PTV volume (cc)	112.6 (578.6)	113.5 (578.6)	110.6 (514.7)	0.91
MD (cm)	4.6 (9.4)	4.5 (9.3)	4.8 (9.2)	0.52
ED (cm)	3.7 (8.4)	3.7 (7.4)	3.8 (8.4)	0.87
LCR (%)	26.2 (84.6)	25.8 (84.6)	26.9 (44.3)	0.59
BEDPTV_max_ (Gy_10_)	51.2 (59.7)	51.4 (57.4)	50.9 (56.8)	0.86
Fractional dose (Gy)	10.4 (22.0)	10.3 (22.0)	10.6 (21.0)	0.78
Duration (days)	9.7 (37.0)	10.2 (29.0)	8.8 (37.0)	0.54
3-Year CSS				0.95
Yes	44 (40.7)	30 (40.5)	14 (41.2)	
No	64 (59.3)	44 (59.5)	20 (58.8)	

*SCC* squamous cell carcinoma, *GTV* gross target volume, *PTV* planning target volume, *MD* maximum diameter for the tumor, *ED* equivalent diameter for the tumor, *LCR* lymphocyte ratio, *BEDPTV*_*max*_ the maximum dose in PTV recorded as BED, *CSS* cancer-specific survival

### Clinical and dosimetric variable screening

Results of univariate and multivariate logistic regression analyses for 3-year CSS was displayed in Table 2. On univariate binary logistic regression analysis, gender, clinical stage, GTV volume, PTV volume, MD, ED, LCR, BEDPTV_max_, fractional dose and treatment duration were significant factors associated with 3-year CSS (*p* < 0.05). However, multicollinearity was detected among GTV volume, PTV volume, MD and ED, in which the tolerance were < 0.1 and a VIF > 10 (Table 3). Therefore, the four factors were excluded from the multivariate analysis. After multivariate binary logistic regression analysis, only gender, clinical stage and LCR were found to be independent factors correlated with 3-year CSS (*p* < 0.05). Female sex was associated improved 3-year CSS (OR 0.04, *p* = 0.008). Patients with advanced stage had a significantly lower 3-year CSS (OR 2.15, *p* = 0.001). Patients with higher LCR levels correlated with a significantly improved 3-year CSS (OR 0.94, *p* = 0.03).

**Table 2:** Results of univariate and multivariate analyses for 3-year CSS

	Univariate analysis		Multivariate analysis	
	*p* value	OR (95% CI)	*p* value	OR (95% CI)
Gender (female vs. male)	0.02	0.08 (0.01–0.67)	0.008	0.04 (0.01–0.44)
Clinical stage (I vs. II vs. III vs. IV)	0.002	1.80 (1.23–2.64)	0.001	2.15 (1.34–3.44)
Histology (adenocarcinoma vs. SCC vs. Unknown)	0.16	1.51 (0.85–2.69)		
GTV volume	0.01	1.01 (1.00–1.02)		
PTV volume	0.008	1.01 (1.0–1.01)		
MD	0.006	1.38 (1.10–1.73)		
ED	0.01	1.49 (1.10–2.01)		
LCR	0.01	0.94 (0.89–0.99)	0.03	0.94 (0.89–1.00)
BEDPTV_max_ (≧ 105.6 vs. < 105.6)	0.01	0.28 (0.11–0.74)		
Fractional dose	0.02	0.88 (0.79–0.98)		
Duration	0.02	1.07 (1.01–1.13)		

*SCC* squamous cell carcinoma, *GTV* gross target volume, *PTV* planning target volume, *MD* maximum diameter for the tumor, *ED* Equivalent diameter for the tumor, *LCR* lymphocyte ratio, *BEDPTV*_*max*_ the maximum dose in PTV recorded as BED

**Table 3:** Results of multicollinearity diagnostics

Variables	Tolerance	VIF
Gender (female vs. male)	0.94	1.07
Clinical stage (I vs. II vs. III vs. IV)	0.72	1.40
GTV volume	0.08	12.85
PTV volume	0.09	10.60
MD	0.10	10.47
ED	0.05	20.32
LCR	0.87	1.15
BEDPTV_max_ (≧ 105.6 vs. < 105.6)	0.51	1.95
Fractional dose	0.42	2.37
Duration	0.46	2.20

*VIF* variance inflation factor, *GTV* gross target volume, *PTV* Planning target volume, *MD* maximum diameter for the tumor, *ED* equivalent diameter for the tumor, *LCR* lymphocyte ratio, *BEDPTV*_*max*_ the maximum dose in PTV recorded as BED

### Radiomics feature selection

**Fig. 1:**
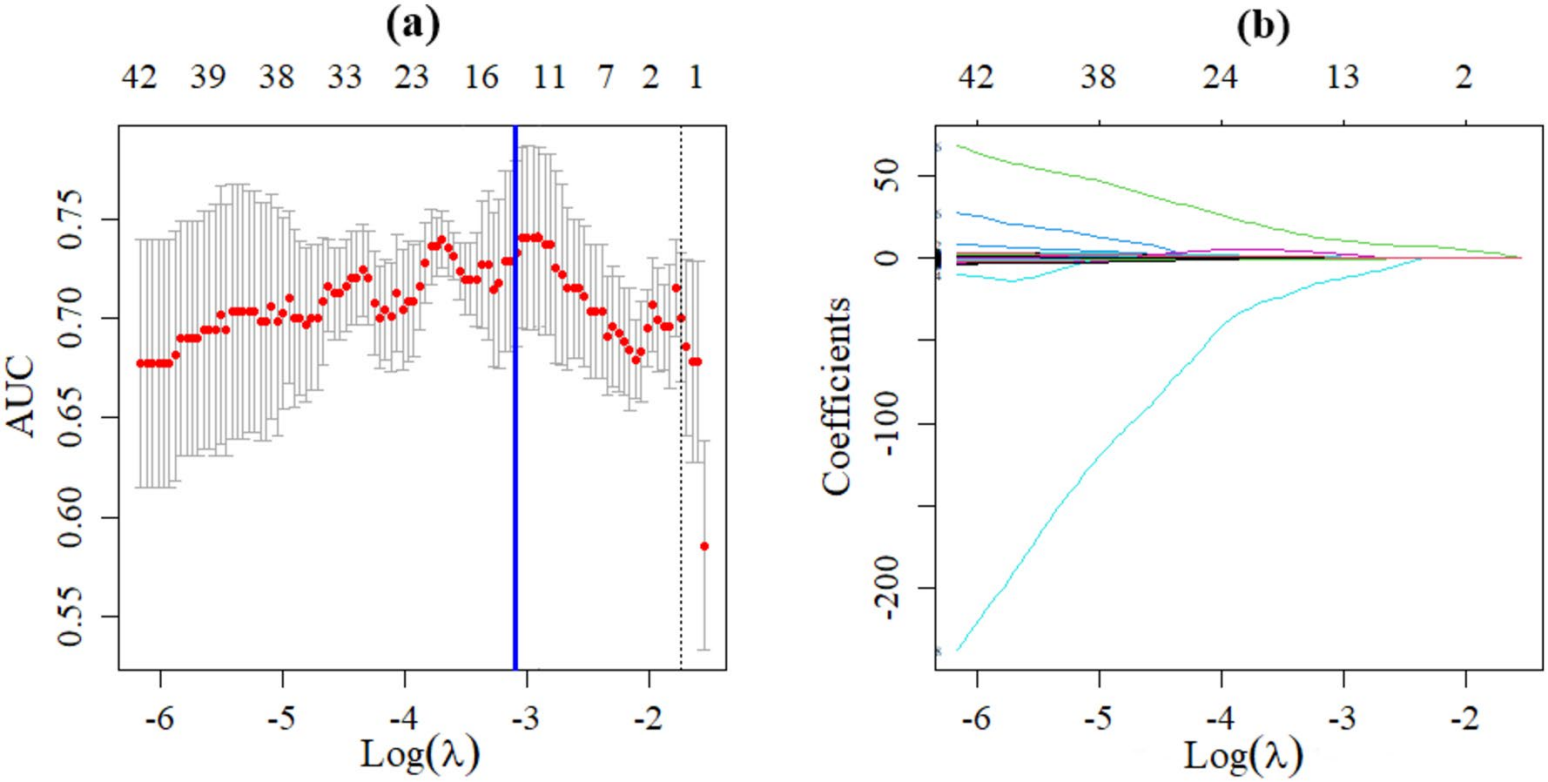
Radiomic variable selection using LASSO regression. **a** The graph of the optimal tuning parameter (*λ*) in the LASSO model. **b** LASSO coefficient profiles of the 13 possible influencing factors

A total of 1502 radiomics features were extracted from the CT images, including 14 shape features, 288 first-order features, and 1200 texture features. 1204 features with good agreement (ICC ≧ 0.75) were included in further analyses. According to the results of univariate analysis, 385 radiomics features were collected, and then 13 potential radiomics features were identified by the LASSO regression. The process of radiomic variable selection using LASSO regression was presented in Fig. 1. Further stepwise regression analysis obtained three derived radiomic features finally, including log.sigma.1.5.mm.3D_glszm_ZoneEntropy, logarithm_ngtdm_Strength, and wavelet.LHL_firstorder_90Percentile. These features were used to calculate the radiomics score using the LR method:

$${\text{Radiomics}} {\text{score}} = {4}0.{685} + {32}.{1}0{7} \times {\text{log}}.{\text{sigma}}.{1}.{5}.{\text{mm}}.{\text{3D}}\_{\text{glszm}}\_{\text{ZoneEntropy}} + {1}.{855} \times {\text{logarithm}}\_{\text{ngtdm}}\_{\text{Strength}} + {3}.{4}0{2} \times {\text{wavelet}}.{\text{LHL}}\_{\text{firstorder}}\_{9}0{\text{Percentile}}$$

### Model performance evaluation

**Fig. 2:**
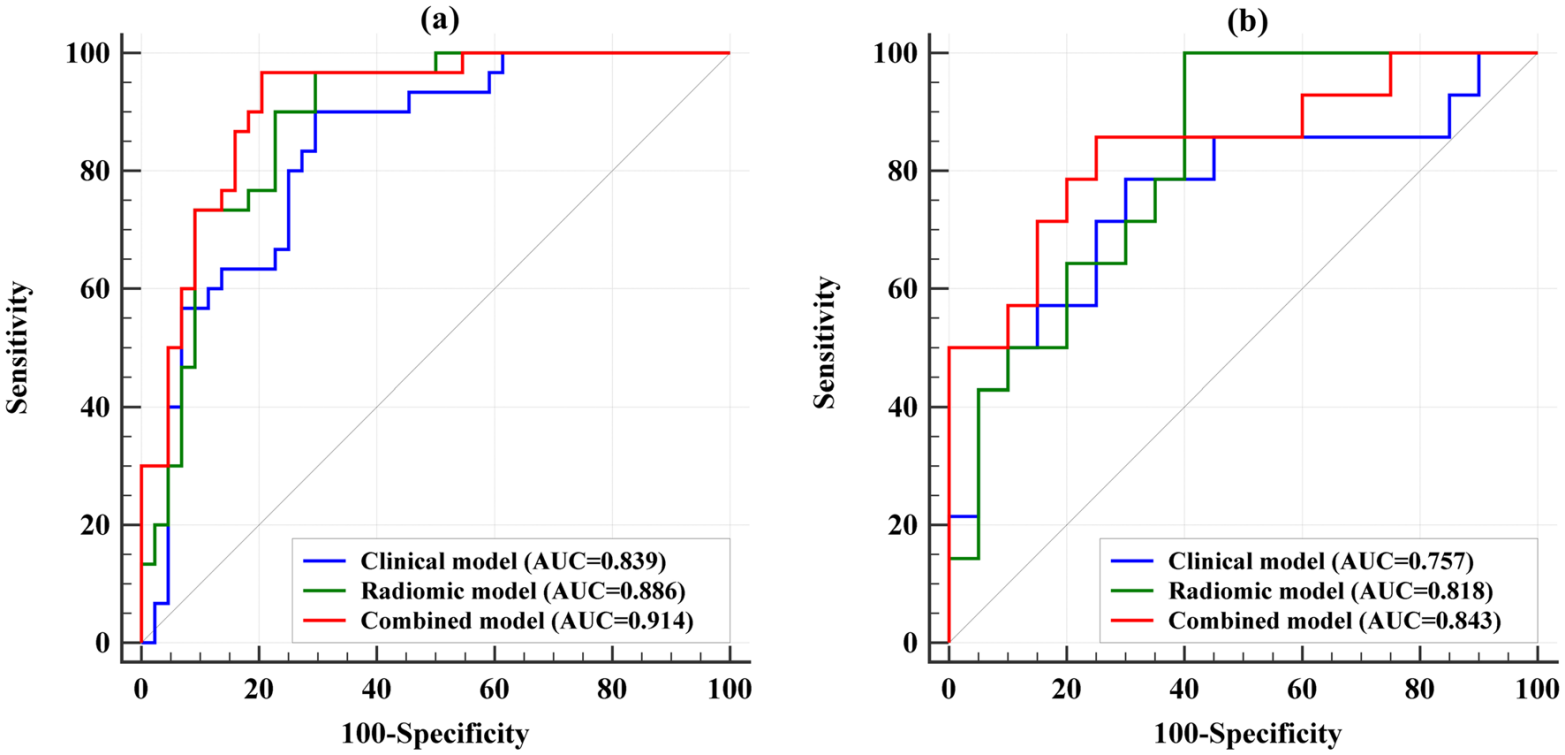
The ROC curves of the clinical, radiomic, and the combined models. **a** Training group. **b** Validation group

**Fig. 3:**
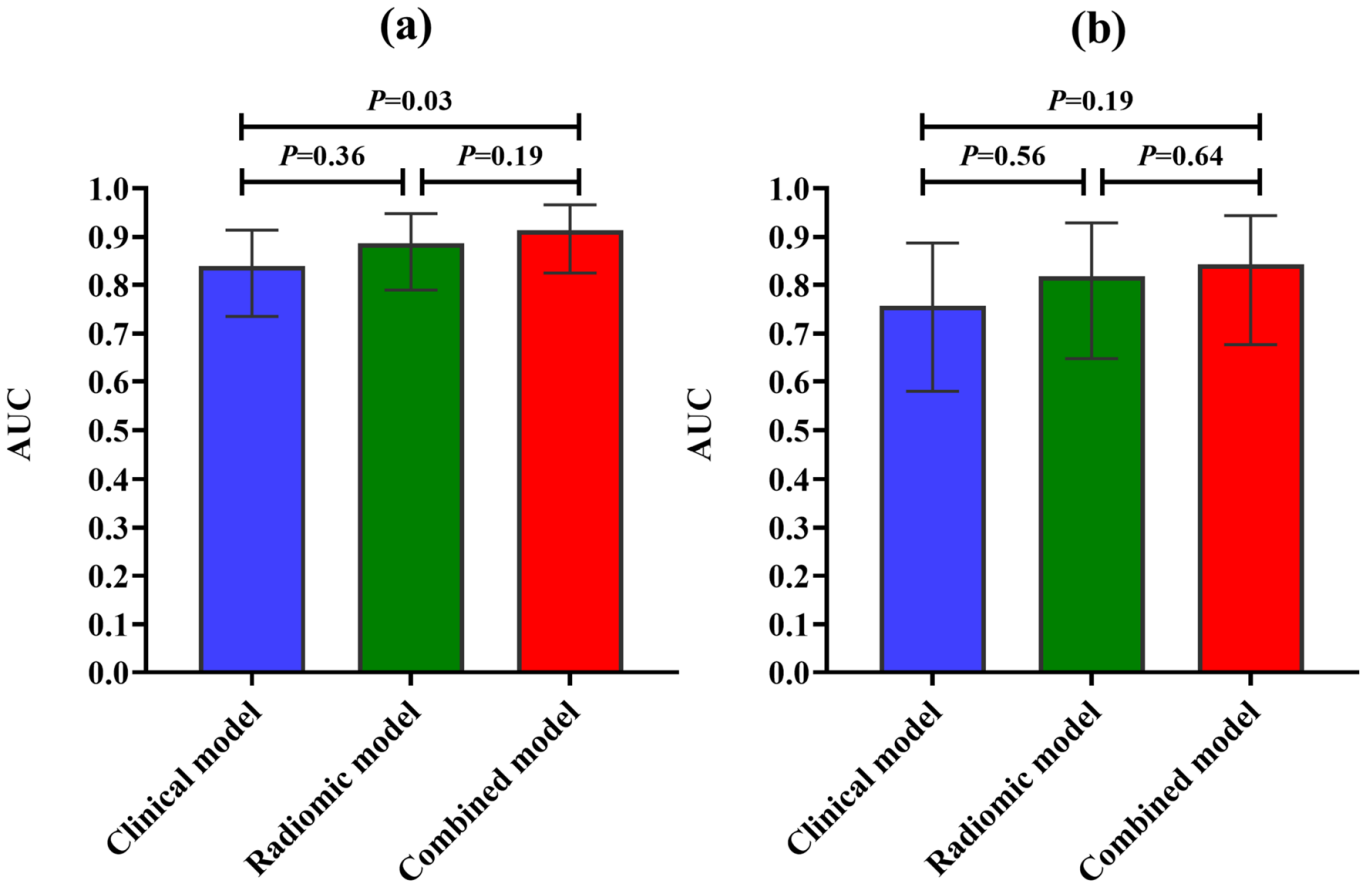
Comparison of AUC values in the clinical, radiomic, and the combined models. **a** Training group. **b** Validation group

**Fig. 4:**
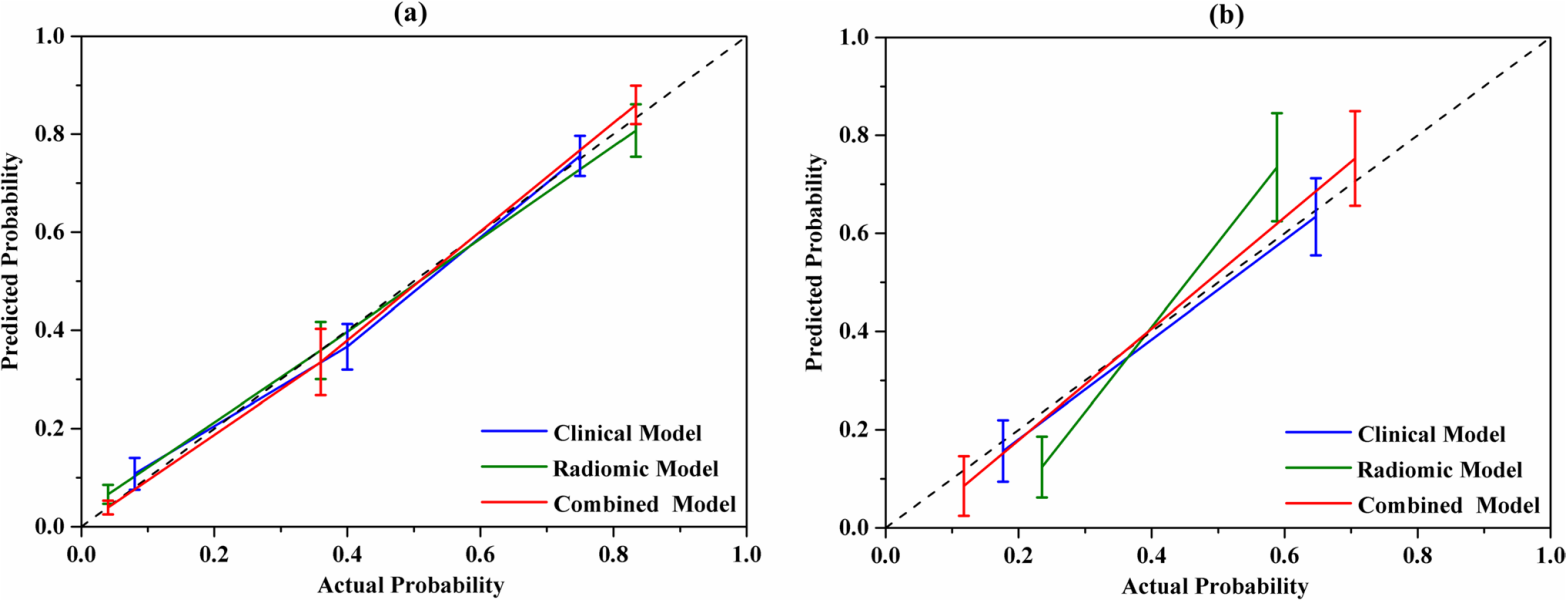
The calibration curves of the clinical, radiomic, and combined models. It is plotted by the actual versus the predicted probabilities. **a** Training group. **b** Validation group

Three prediction models were established: the clinical model, radiomic model and combined model. ROC curves of the three models were displayed in Fig. 2. AUC values of the clinical model, radiomic model, and the combined model were 0.839 (95% CI 0.735–0.914), 0.886 (95% CI 0.790–0.948), and 0.914 (95% CI 0.825–0.966) in the training cohort and 0.757 (95% CI 0.580–0.887), 0.818 (95% CI 0.648–0.929), and 0.843 (95% CI 0.677–0.944) in the validation cohort. The combined model showed superior discriminative ability compared to the clinical models in the training cohort (*p* < 0.05). However, there was no statistical significance among the three models in the validation cohort (Fig. 3). The accuracy, sensitivity, and specificity values of these models were presented in Table 4. The calibration curves for the models were shown in Fig. 4. The results confirmed that the predicted probability of 3-year CSS were consistent with the actual observation with *p* > 0.05 in the Hosmer–Lemeshow goodness-of-fit test (Table 5).

**Table 4:** Performance of the clinical, radiomic and the combined models

Endpoint	Group	Models	AUC (95% CI)	*p* value	Accuracy (%)	Sensitivity (%)	Specificity (%)
3-Year CSS	Training	Clinical model	0.839 (0.735–0.914)	< 0.001	78.4	70.5	90.0
		Radiomic model	0.886 (0.790–0.948)	< 0.001	81.1	90.0	77.3
		Combined model	0.914 (0.825–0.966)	< 0.001	86.5	96.7	79.5
	Validation	Clinical model	0.757 (0.580–0.887)	0.005	73.5	78.6	70.0
		Radiomic model	0.818 (0.648–0.929)	< 0.001	76.5	100.0	60.0
		Combined model	0.843 (0.677–0.944)	< 0.001	79.4	85.7	75.0

The criterion values recommended by the MedCalc software for accuracy calculation for the clinical, radiomic, and combined model were 0.2865, 0.3324, and 0.2737 in the training group and 0.3625, 0.2101, and 0.4332 in the validation group, respectively

*CSS* cancer-specific survival

**Table 5:** Hosmer–Lemeshow goodness-of-fit test for the clinical, radiomic and the combined models

Endpoint	Group	Models	Chi-square	*p* value
3-Year CSS	Training	Clinical model	4.79	0.31
		Radiomic model	9.66	0.29
		Combined model	9.42	0.31
	Validation	Clinical model	5.81	0.67
		Radiomic model	9.83	0.28
		Combined model	8.33	0.40

*CSS *cancer-specific survival

### Nomogram establishment

**Fig. 5:**
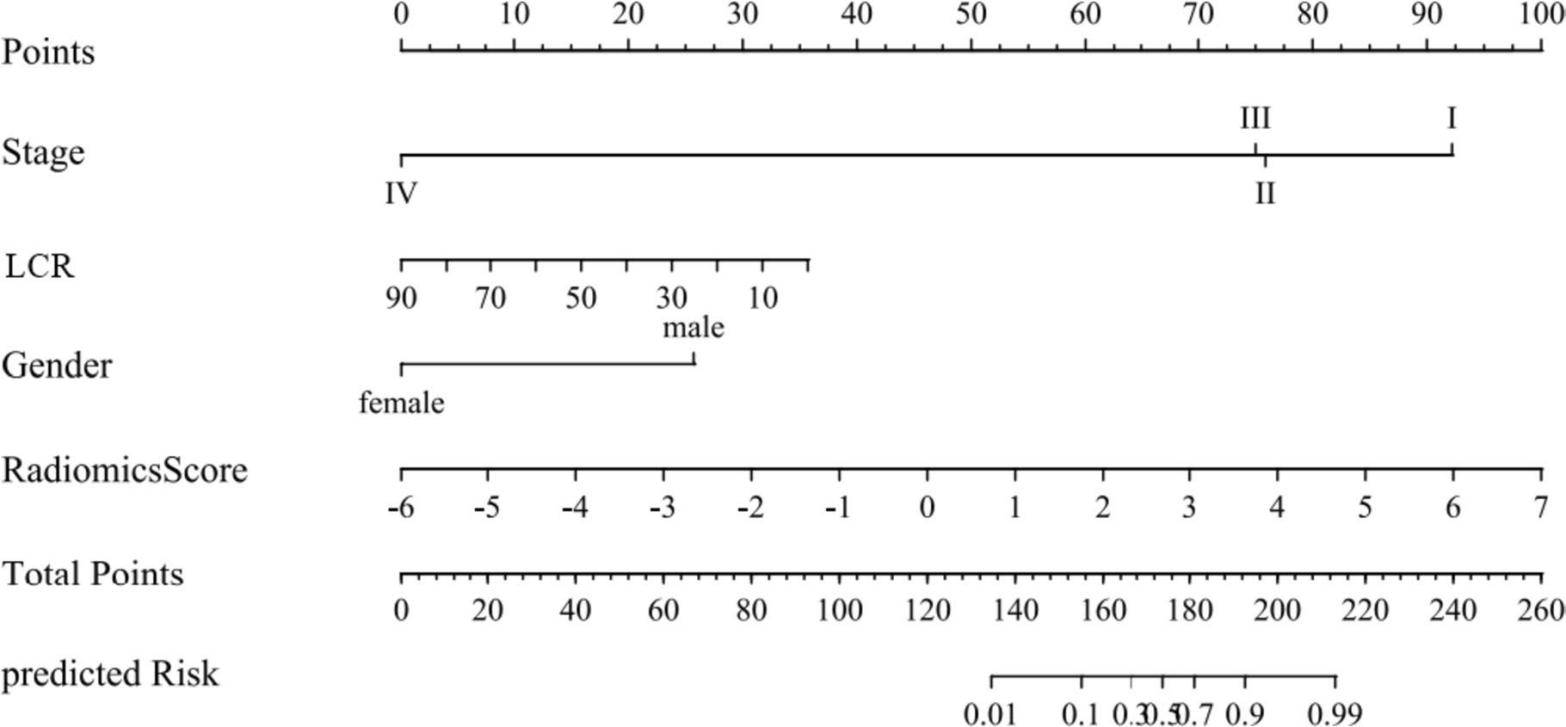
The nomogram for prediction 3-year CSS in lung cancer patients after SBRT. *LCR *lymphocyte ratio

**Fig. 6:**
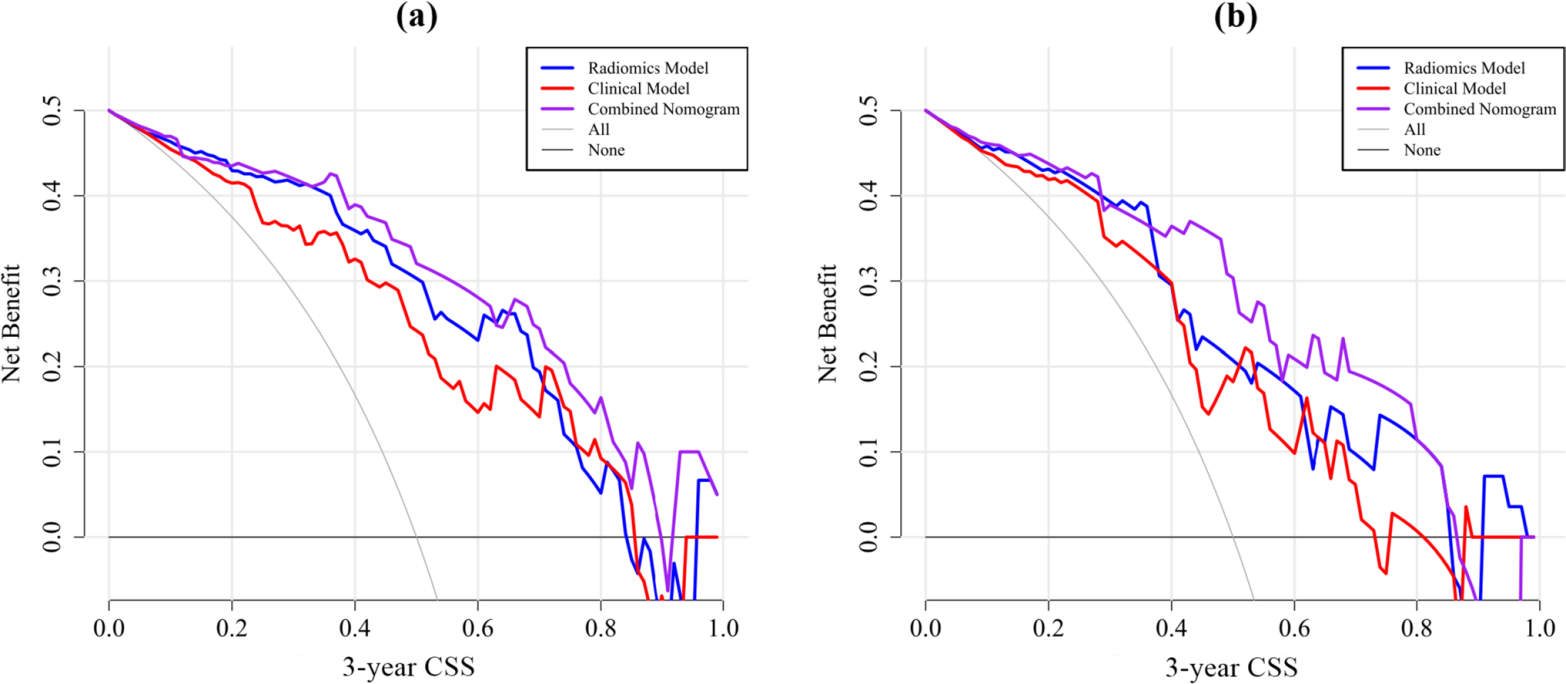
Decision curves for clinical model, radiomic model and the combined model for 3-year CSS after SBRT. **a** Training group. **b** Validation group. *CSS *cancer-specific survival

A nomogram based on the combined model was constructed by considering gender, clinical stage, LCR, and the radiomics score factors (Fig. 5). The decision curve revealed that the clinical model, the radiomic model, and the combined nomogram were all beneficial for predicting 3-year CSS. The area under the curve of the combined nomogram was larger than that of clinical and radiomic models, indicating that the combined nomogram had the highest potential for clinical application (Fig. 6).

## Discussion

In the study, we identified three clinical factors associated with 3-year CSS for lung cancer patients treated with SBRT: gender, clinical stage, and LCR. We also found three radiomic features associated with 3-year CSS: log.sigma.1.5.mm.3D_glszm_ZoneEntropy, logarithm_ngtdm_Strength and wavelet.LHL_firstorder_90Percentile. Importantly, we demonstrated that a model combining clinical parameters with radiomic features could predict the 3-year CSS effectively. To our knowledge, this is the first study to integrate clinical and radiomic signatures to predict 3-year CSS for lung cancer patients treated with SBRT. Based on our findings, radiation oncologists should consider these factors when predicting CSS in lung cancer patients receiving SBRT.

In the era of personalized medicine, accurately predicting prognosis is essential for guiding individualized clinical decision-making (Kang et al. 2020). Moreover, improved survival prediction and risk stratification in NSCLC would benefit prognosis counseling, adjuvant therapy selection, and clinical trial design (Somasundaram et al. 2023). Therefore, the prediction model developed in the study has the potential to identify lung cancer patients most likely to benefit from SBRT treatment and guide treatment strategy.

Three clinical parameters including gender, clinical stage and LCR were found to be independent prognostic factors for 3-year CSS in lung cancer patients who underwent SBRT. The finding is consistent with published literature that indicates female sex as an independent and favorable prognostic factor in patients treated with SBRT. Kang et al. reported that female gender was associated with 5-year survival for patients after SBRT (Kang et al. 2020), and similar results were also confirmed in Baker’s research, in which female was found to be significant predictors of 6-month, 1-year, 3-year and 5-year survival in the early NSCLC population following SBRT (Baker et al. 2020). Jacobs et al. also demonstrated that female sex was one of the factors strongly associated with improved OS on multivariable analysis (HR = 0.81) (Jacobs et al. 2022). Furthermore, clinical stage is recognized as the most important variable associated with OS for lung cancer (Amin et al. 2018). Recently, the prognostic role of blood inflammation parameters has been getting increasing attention in the immunotherapy era. Sebastian et al. reported that the pre-treatment neutrophil–lymphocyte ratio (NLR) was associated with mortality in patients treated with SBRT in two other institutions (Sebastian et al. 2019). Afterwards, this finding was confirmed in single and multi-fraction SBRT for early-stage lung cancer (Dong et al. 2023; Huang et al. 2023; Kotha et al. 2021). However, evidence regarding the prognostic value of LCR in lung SBRT is rather scarce. To our knowledge, we are the first to report that LCR is associated with 3-year CSS, with higher LCR levels correlating with improved outcomes. It is worth noting that future studies are expected to confirm the role of LCR levels in correlation with 3-year CSS and elucidate the underlying immune mechanism behind.

Radiomic features extracted from CT images have shown promising performance in predicting OS in patients with NSCLC in various studies. Huynh found that two radiomic features, namely LoG 3D run low gray level short run emphasis and stats median, were associated with OS (Huynh et al. 2016). Franceschini et al. successfully predicted disease-specific survival in patients treated with SBRT using four radiomic features (Franceschini et al. 2020). Sawayanagi et al. developed an OS prediction model for primary NSCLC after SBRT through radiomics analysis (Sawayanagi et al. 2022). Jiao et al. also developed a radiomic model to integrate risk of death estimates based on pre- and post-treatment CT scans in patients receiving SBRT (Jiao et al. 2021). In our study, we also identified three derived texture features associated with 3-year CSS, namely log.sigma.1.5.mm.3D_glszm_ZoneEntropy, logarithm_ngtdm_Strength, and wavelet.LHL_firstorder_90Percentile. However, there is currently no consensus on a specific radiomic biomarker, partially due to reported variations in CT acquisition parameters, reconstruction techniques, radiation dose, and reconstruction settings, which can impact the reproducibility of radiomic features (Berenguer et al. 2018; Meyer et al. 2019). Thus, it is essential to standardize the extraction of radiomic features and follow reporting guidelines (Lambin et al. 2017). Recently, some radiomic features have been used to predict the tumor stage (Nie et al. 2023; Demirjian et al. 2022; Sun et al. 2018). Therefore, we infer there might be some intrinsic connection between the radiomic features and tumor stage for lung cancer patients. It is worth noting that future studies should aim to validate the role of the radiomic predictors proposed in this study.

In this current study, we found the model incorporating both clinical and radiomic parameters performed better in the training cohort than the clinical model alone. However, we did not observe a similar trend in the validation cohort due to limited sample size. This finding is consistent with other studies (Luo et al. 2021; Zhai et al. 2020, 2017) and the result indicates that multi-omics features contribute to improve the prediction accuracy of radiation therapy models (Cui et al. 2021).

There are several limitations to this study. First, the proposed model was developed from a single-institution retrospective database and would benefit from external validation at other institutions. Second, the inclusion of heterogeneous stage lung cancer patients may have weaken the results. Third, the study had limited sample size. The sample size was too small to detect any predictive improvement using the clinical-radiomic over the clinical model. According to Vittinghoff et al., logistic and Cox models may achieve acceptable results with 5–9 events per variable (EPV) in a range of circumstances (Vittinghoff and McCulloch 2007). Therefore, we should have included no more than 6 variables in the univariate analysis using the 5 EPV rule with minimum sample size. However, we employed 11 variables to account for any potentially clinically significant variables. Despite this, we believe the model was successfully established for the following reasons: (1) The OR values and the confidence interval coverage in the study were within normal ranges. (2) The significant variables identified in the multivariate analysis were consistent with previous studies. Fourth, it is worth noting that manual tumor segmentation for radiomics analysis is time-consuming and labor-intensive (Tsai et al. 2022), which may limit its clinical usefulness. Finally, since radiomics features are potentially dependent on imaging quality and incorporating clinical and radiomic signature into one nomogram might reduce the robustness of the model.

## Conclusion

Three clinical factors, including gender, clinical stage and LCR, as well as three radiomic features, were found to be independent factors correlated with 3-year CSS. A combined model that integrates clinical and radiomic parameters was developed to predict 3-year CSS prediction for lung cancer patients after SBRT.

## Data Availability

The datasets generated during and/or analysed during the current study are available from the corresponding author on reasonable request.
